# siRNA-E6 sensitizes HPV-16-related cervical cancer through Oxaliplatin: an in vitro study on anti-cancer combination therapy

**DOI:** 10.1186/s40001-023-01014-9

**Published:** 2023-01-21

**Authors:** Parisa Shiri Aghbash, Nima Hemmat, Behzad Baradaran, Hossein Bannazadeh Baghi

**Affiliations:** 1 Immunology Research Center, Tabriz University of Medical Sciences, Tabriz, Iran; 2 Department of Virology, Faculty of Medicine, Tabriz University of Medical Sciences, Tabriz, Iran; 3 Department of Immunology, Faculty of Medicine, Tabriz University of Medical Sciences, Tabriz, Iran; 4 Infectious and Tropical Diseases Research Center, Tabriz University of Medical Sciences, Tabriz, 5166/15731 Iran

**Keywords:** Cervical cancer, Combination therapy, E6 oncoprotein, Oxaliplatin, siRNA

## Abstract

**Background:**

Persistent infection with high-risk Human papillomaviruses (HPV), such as hr-HPV-16 and hr-HPV-18, lead to cervical cancer, the fourth most common cancer in the world. In the present study, we investigated the alteration of E6 oncogene expression by E6-specific short interfering RNA (siRNA) combined with Oxaliplatin.

**Methods:**

The cervical cancer cell line, CaSki, was transfected with E6-siRNA, then treated with Oxaliplatin. The cellular genes, such as *p53*, *MMP9*, *Nanog,* and caspases expression, were assessed by quantitative real-time PCR. The cell death rate, cell cycle, and cell viability were assessed by Annexin V/PI staining, DAPI staining, and MTT test, respectively. Furthermore, colony formation assay and scratch test determined the stemness ability and cell metastasis, respectively.

**Results:**

Combination therapy increased the re-expression of genes involved in the p53-dependent apoptosis pathway (increase in apoptosis to 44.2%), and reduced stemness and metastasis ability compared to either siRNA or Oxaliplatin monotherapy. Together, our results demonstrate that E6-siRNA and Oxaliplatin combination increased the cervical cancer cells’ sensitivity to Oxaliplatin and decreased the survival rate, proliferation, and metastasis, and consequently escalated apoptosis rate, induced cell cycle arrest in the sub-G1 stage, and reduced the chemotherapy drug dosage.

**Conclusion:**

Inhibition of E6 oncogene expression and subsequent E6-siRNA with Oxaliplatin combination therapy could be a novel strategy for cervical cancer treatment.

**Graphical Abstract:**

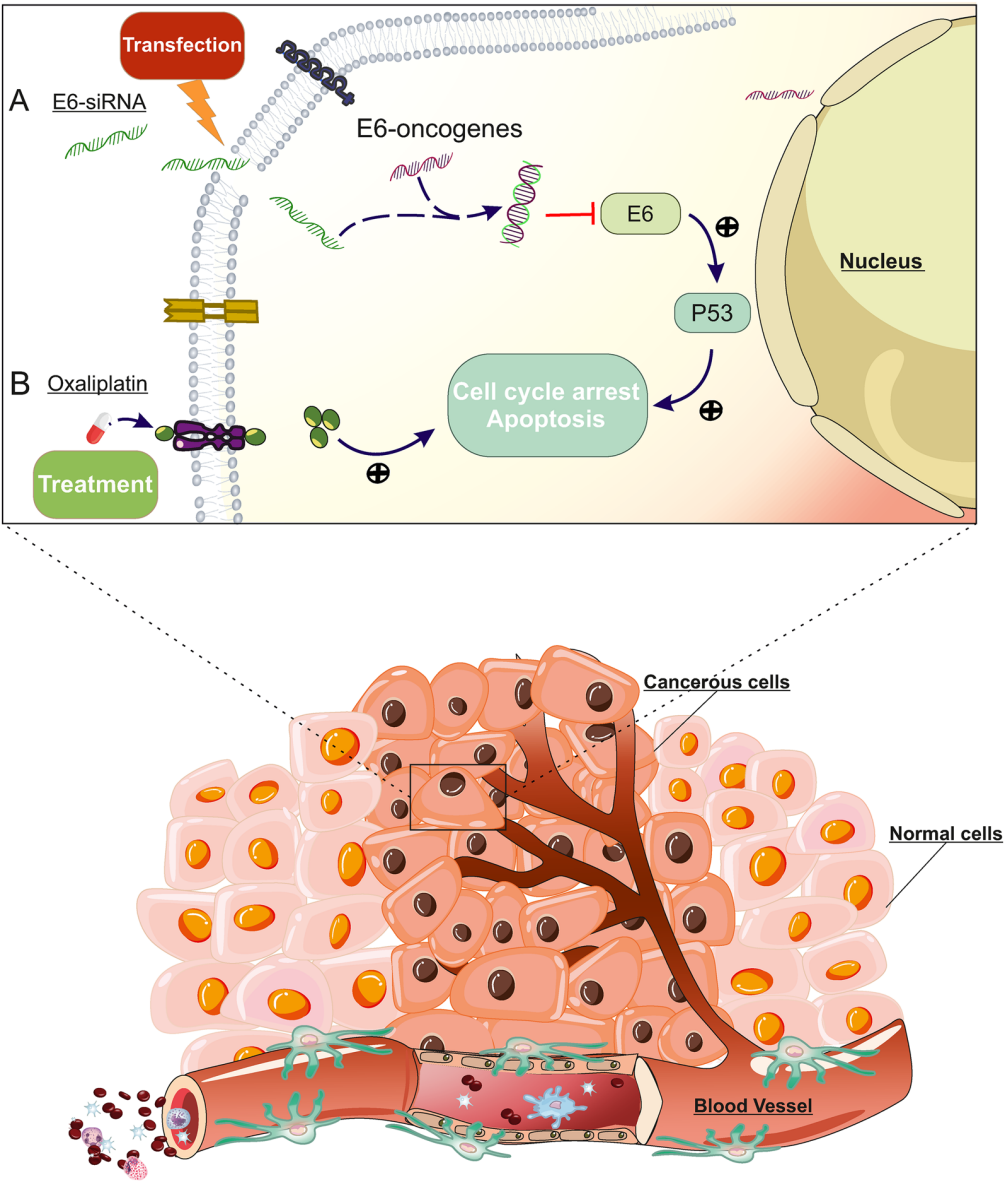

## Background

In 1983, Harald Zur Hausen et al. first showed the link between human papillomaviruses (HPV) and related cancers, especially cervical cancer [1]. Cervical cancer is the fourth most common cancer in women; in 2020, it accounted for about 342,000 deaths and 604,000 new cases [2]. Moreover, according to the World Cancer Reports, the development of cervical cancer is caused by persistent infection of epithelial cells with one or more types of HPV, leading to precancerous lesions and resulting in cervical malignancy [3]. So far, about 200 types of HPV have been identified, some of which are carcinogenic; they are divided into low-risk, likely high-risk, and high-risk types based on their carcinogenicity and severity of clinical symptoms [4, 5]. In this regard, reports indicated that about 100% of cervical cancers are due to infection with high-risk types of HPV, of which both HPV-16 and HPV-18 account for 70% of cases [6]. Early proteins of HPVs are expressed following viral infection in infected cells and are associated with disorders [3]. Early oncoproteins, such as E5, E6, and E7, have participated in immortality and uncontrolled cell proliferation and interfere with regulating the cell cycle and signaling pathways for the repair and apoptosis of infected cells [7]. For instance, the E6 protein acts as an allosteric activator of E6-associated protein (E6AP) function and increases E6AP accumulation [8]. Notably, the E6 protein exerts its carcinogenic effect by suppressing the apoptotic process of the p53 protein and, eventually, the p53 level reduction [3]. In addition, E6 oncoprotein binds to other cellular pro-apoptotic proteins, such as BAK, FADD, procaspase-8, or c-MYC, inducing their proteolytic degradation in an E6AP-dependent manner [9]. However, the exact mechanisms for regulating the E6/E6AP/p53 complex are unknown. Many efforts have been made to investigate the HPV’s role in cervical cancer progression and develop new diagnostic, prevention, and treatment methods for cervical malignancy.

Oxaliplatin, as a platinum compound, has shown a broad range of anti-cancer activities in in vitro systems and preclinical and clinical investigations. Oxaliplatin has significant cytotoxic, pharmacological, and biochemical characteristics contribute to drug resistance via various pathways [10]. These treatments for cervical cancer can be used in individuals or in combination with other therapies. Therefore, due to the great attention of scientists to gene therapy, in recent years, many efforts have been made to develop gene therapy strategies for cancer treatment [3]. It has been reported that clinical trials associated with gene therapy have led to treating 65% of cancers. For the first time in 2001, the effect of gene extinguishing on mammalian cells was reported, in which small interfering RNA (siRNA) was considered an innovative tool for gene therapy. Indeed, siRNAs with a length of 20–25 base pairs could lead to regulating the specific gene expression and subsequently gene silencing. Due to the complexity of cancer, the combination of gene therapy and chemotherapy as an emerging therapy to overcome chemical resistance and access to synergistic cancer treatment has been considered in recent years [11]. Reports indicate that the silencing of E6/E7 genes can lead to the induction of apoptosis in cervical cancer cell lines. Thus, combining gene therapy with chemotherapy by targeting HPV E6/E7 genes can increase antitumor activity, induce synergistic toxicity and cellular sensitivity, and reduce the effective dose of chemotherapy and unwanted toxicity [12]. This study aims to investigate the E6-siRNA effects on synergistic toxicity, reduce the effective dose of chemotherapy, improve the therapeutic effect, and reduce the local and systemic toxicity against cervical cancer.

## Methods

### Cell culture

The human cervical cancer cell line CaSki was purchased from the Iranian Biological Resource Center (IBRC); these epithelial carcinoma cells contain ~ 600 copies of the integrated HPV-16 genome. The cells were cultured as monolayers and maintained in a culture medium. The culture medium was Roswell Park Memorial Institute medium (RPMI 1640; Gibco, USA) containing 10% Fetal Bovine Serum (FBS; Gibco, USA), 100 U/ml Penicillin, and 100 U/ml Streptomycin; all gathered in a T75 flask. The cultured cells were incubated in 5% CO_2_, 95% humidity, and 37 °C conditions. When the confluency of cells reached 70%, the cells were detached by 0.25% Trypsin‐EDTA (Gibco, USA) for cell passage.

### Transfection of E6-siRNA

E6-siRNA was purchased from Santa Cruz, United States. To determine the effective dose of siRNA, it was transfected to the CaSki cell line with a series of different doses by using Gene Pulser (Bio-Rad, USA) electroporation system. To ensure siRNA specificity, it was evaluated by Nucleotide BLAST. It was shown that the siRNA effectively influenced all HPV-16 strains and E6 gene transcripts. After being affected by the electroporation method (length = 8 ms and voltage = 130 V), the cells were seeded on different plates according to the type of test. The final concentration of siRNA during transfection was 100 pmol.

### MTT assay

3-(4,5‐dimethylthiazol-2‐yl)‐2,5‐diphenyltetrazolium bromide (MTT) assay was used to assess cell viability. This way, about 1.5 × 10^4^ cells per well were seeded into the 96-well plate. The cells were divided into two groups, cells in the normal group were not transfected with E6-siRNA and the E6-siRNA transfected cells. Both groups were treated with Oxaliplatin, a water-soluble drug stored at room temperature and away from direct light. Different concentrations of the drug were then replaced with cells’ supernatant that had been seeded in 96-well plates 24 h earlier. After 24 h incubation, the medium was discarded and the 150 µl MTT solution (2 mg/ml) was added to each well; it was incubated for 4 h at 37 °C and 5% CO_2_. The supernatant was discarded and to dissolve the formed formazan crystals, 150 μl of dimethyl sulfoxide (DMSO) was added to each well; then, the absorption was read at 570 nm wavelength and 620 nm for reference wavelength with a microplate reader (Tecan, Switzerland). All tests were performed in triplicate.

### Wound healing assay (scratch test)

Cell migration ability of CaSki cells after transfection of E6-siRNA and treatment with Oxaliplatin was measured by wound healing assay. For this purpose, transfected and non-transfected cell groups were seeded in 24-well plates as a single layer and incubated for 24 h at 37 °C and 5% CO_2_. Before treatment, the plate divided into four groups: control, E6-siRNA transfected cells, Oxaliplatin treated cells, and combination of Oxaliplatin and E6-siRNA. After 24 h of siRNA transfection and cells' seeding, a linear wound was created on the cell monolayer by the 100 pipettes tip; then, the related groups were treated with IC_25_ concentration of Oxaliplatin. Cells of each group were examined and captured by utilizing an inverted microscope (Optika, XDS‐3, Italy) for determining migration in 0 h, 24 h, 48 h, and 72 h.

### Quantitative real-time PCR (qRT-PCR)

For this test, 3 × 10^5^ CaSki cells in transfected and non-transfected groups were seeded into a 6-well plate and divided into four groups––control, E6-siRNA transfected, Oxaliplatin treated, and combination of Oxaliplatin and E6-siRNA––then incubated for 24 h at 37 °C and 5% CO_2_. Next, related groups were exposed to appropriate doses of Oxaliplatin (IC_25_) for 24 h. According to the protocol, total RNA was extracted from the cells using TRIzol reagent (RiboEX, South Korea). Then a Nano-drop device (Thermo Scientific, USA) with wavelengths of 230, 260, and 280 nm was used to assess the concentration and purity of the extracted RNA. Following that, 2-Step 2X RT-PCR Pre-Mix (BioFACT^™^, South Korea) was used to synthesize complementary DNA (cDNA) to measure the relative expression of related genes at 1000 ng/μl of total RNA. The expression of the *CD133*, *CD44*, *Nanog*, *BAX*, *BCL2*, *Casp-3*, *Casp-8*, *Casp-9*, *MMP9*, *p53*, and *HPV-16 E6* was evaluated using the 2X Real-Time PCR Master Mix (BioFACT^™^, South Korea) and quantified by the StepOnePlus Real-Time PCR system (Applied Biosystems, Foster City, USA). GAPDH was used as an internal control for normalization and gene expression analysis and function 2^−ΔΔCT^ analysis. It is worth noting that all primer pairs sequences were blasted using the NCBI’s Primer-BLAST tool before the experiment. The sequences of the primer pairs are shown in Table 1.

**Table 1:** The primer pairs sequences used for qRT-PCR

Gene name	Forward	Reverse
E6	5′-AAGCAACAGTTACTGCGACG-3′	5′-GGACACAGTGGCTTTTGACA-3′
CD133	5′-GACCGACTGAGACCCAACATC-3′	5′-GGCTAGTTTTCACGCTGGTCA-3′
CD44	5′-CCAGAAGGAACAGTGGTTTGGC-3′	5′-ACTGTCCTCTGGGCTTGGTGTT-3′
Nanog	5′-AGAGGTCTCGTATTTGCTGC-3′	5′-ACACTCGGTGAAATCAGGGTA-3′
BAX	5′-TTTGCTTCAGGGTTTCATCCA-3′	5′-TCTGCAGCTCCATGTTACTGTC-3′
BCL2	5′-CTGTGGATGACTGAGTACCTG-3′	5′-GAGACAGCCAGGAGAAATCA-3′
Casp-3	5′-CAAACCTCAGGGAAACATTCAG-3′	5′-CACACAAACAAAACTGCTCC-3′
Casp-8	5′-CGGACTCTCCAAGAGAACAGG-3′	5′-TCAAAGGTCGTGGTCAAAGCC-3′
Casp-9	5′-CTGTCTACGGCACAGATGGAT-3′	5′-GGGACTCGTCTTCAGGGGAA-3′
MMP9	5′-ATTCATCTTCCAAGGCCAATCC-3′	5′-CTTGTCGCTGTCAAAGTTCG-3′
P53	5′-AAAGTCTAGAGCCACCGTCC-3′	5′-AATCCAGGGAAGCGTGTCA-3′

### Apoptosis assay

In the beginning, 3 × 10^5^ CaSki cells in two transfected and non-transfected groups were seeded into a 6-well plate. Before treatment the plate was divided into four groups: control, E6-siRNA transfected cells, Oxaliplatin treated cells, and combination of Oxaliplatin and E6-siRNA. After 24 h, related groups were treated with IC_25_ concentration of Oxaliplatin. The Annexin V/PI staining kit (Exbio–Czech) was used to prepare cells for flow cytometry. For this purpose, after 24 h incubation, the cells were first washed with 1X PBS and then stained with FITC-labeled Annexin V and PI according to the kit protocol. Finally, apoptotic cells were evaluated by flow cytometry.

### Cell cycle assay

After transfection, 3 × 10^5^ CaSki cells were seeded in 6-well plates in two transfected and non-transfected groups. They were categorized into four groups after 24 h of incubation: control, E6-siRNA transfected, Oxaliplatin treated, and combination of Oxaliplatin and E6-siRNA. Next, the cells were prepared for examination after transfection and treatment of the target groups with an IC_25_ concentration. For this purpose, the cells were washed with PBS, fixed with cold 70% ethanol, and stored overnight at − 20 °C. Then, the cells were re-suspended in PBS containing RNase A (200 g/ml), incubated at 37 °C for 30 min, and stained with DAPI (50 g/l) for analysis. Flowingly, flow cytometry (Milteny Biotec MACSQuant 10) was used to assess the distribution of cells at each phase of the cell cycle, and the data were processed using FlowJo FACS software.

### Colony formation assay

To execute the colony formation test, 10^4^ CaSki cells per well were seeded into a 6-well plate in two transfected and non-transfected groups and divided into four groups after 24 h of incubation: control, E6-siRNA transfected, Oxaliplatin treated, and combination of Oxaliplatin and E6-siRNA. After a 24 h incubation at 37 °C and 5% CO_2_, the target groups were given an effective dose of Oxaliplatin. Then, every 2 to 3 days, the medium in each well was replaced, and the colonies were identified 10 to 12 days later. After that, the colonies were stained with crystal violet and maintained to incubate for 30 min. Finally, ImageJ software was used to count the number of colonies in each group.

### Statistical analysis

The significance of data was assessed using one-way and two-way ANOVA in GraphPad Prism v8 (San Diego, California, USA, http://www.graphpad.com) as well as Tukey’s multiple comparison test. In the beginning, all data are presented as mean ± SD, with a *p*-value of 0.05 providing the significance cut-off criterion.

## Results

### Optimization of time-dependent and dose-dependent reduced expression of E6

**Fig. 1:**
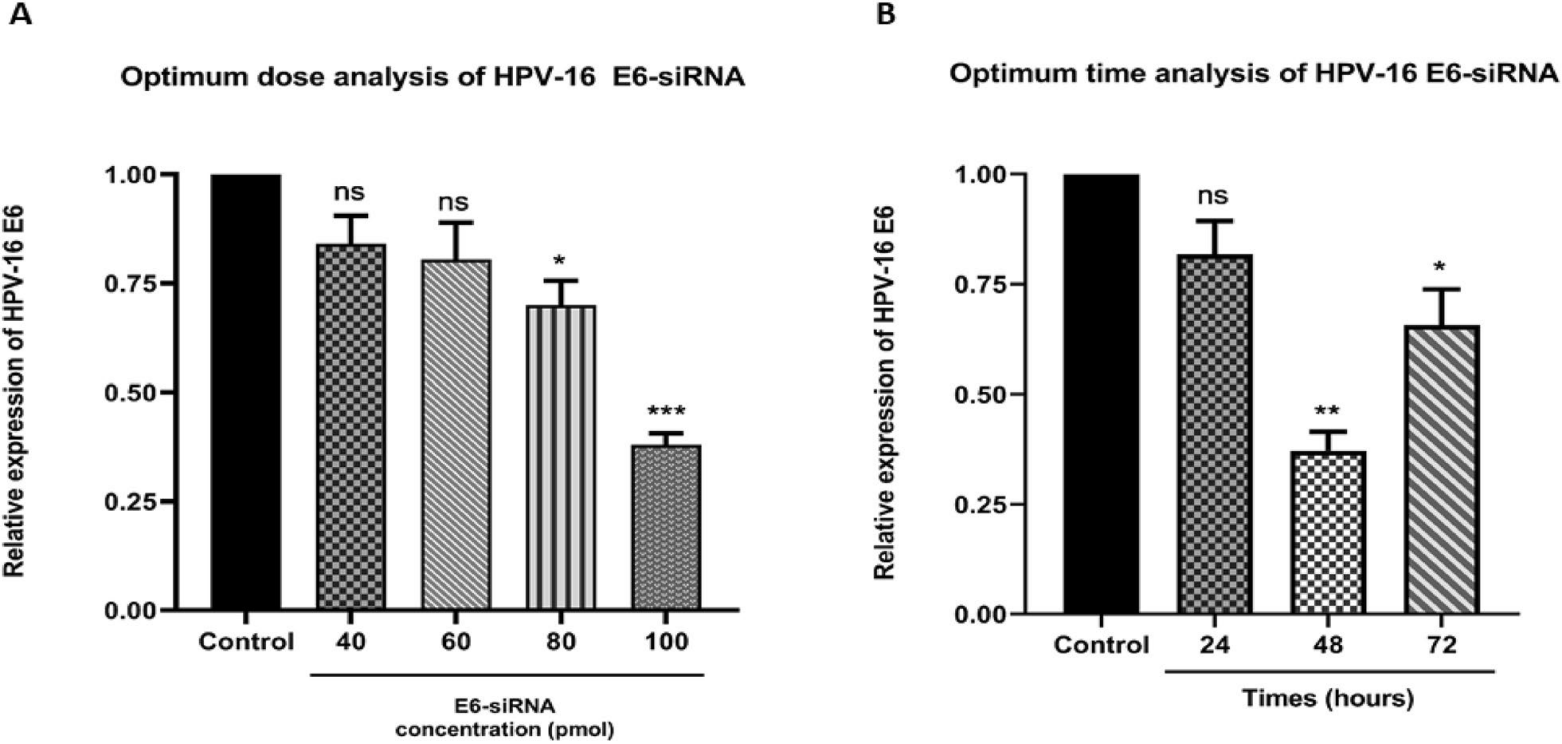
**A**. The amount of E6 oncogene expression in the CaSki cell line showed the most significant reduction in the gene expression at a concentration of 100 pmol. **B**. E6-siRNA has been proven to decrease E6 oncogene expression in 48 h. The dose of 100 pmol should be determined as the optimum dose in 48 h (*p < 0.05, **p < 0.01, ***p < 0.001)

To evaluate the activity of HPV-16 E6 oncogene, CaSki cells were transfected with several doses of siRNA (40, 60, 80, and 100 pmol) to identify the effective dose of siRNA at an adequate time. Cells transfected with 100 pmol of E6-siRNA were demonstrated to dramatically lower E6 oncogene expression, and also 48 h was confirmed to be the best period for siRNA activity, according to the qRT-PCR measurement of mRNA E6 expression (Fig. 1A, B).

### Monotherapy with Oxaliplatin or E6-siRNA has a slight effect on cell viability compared to combination therapy

**Fig. 2:**
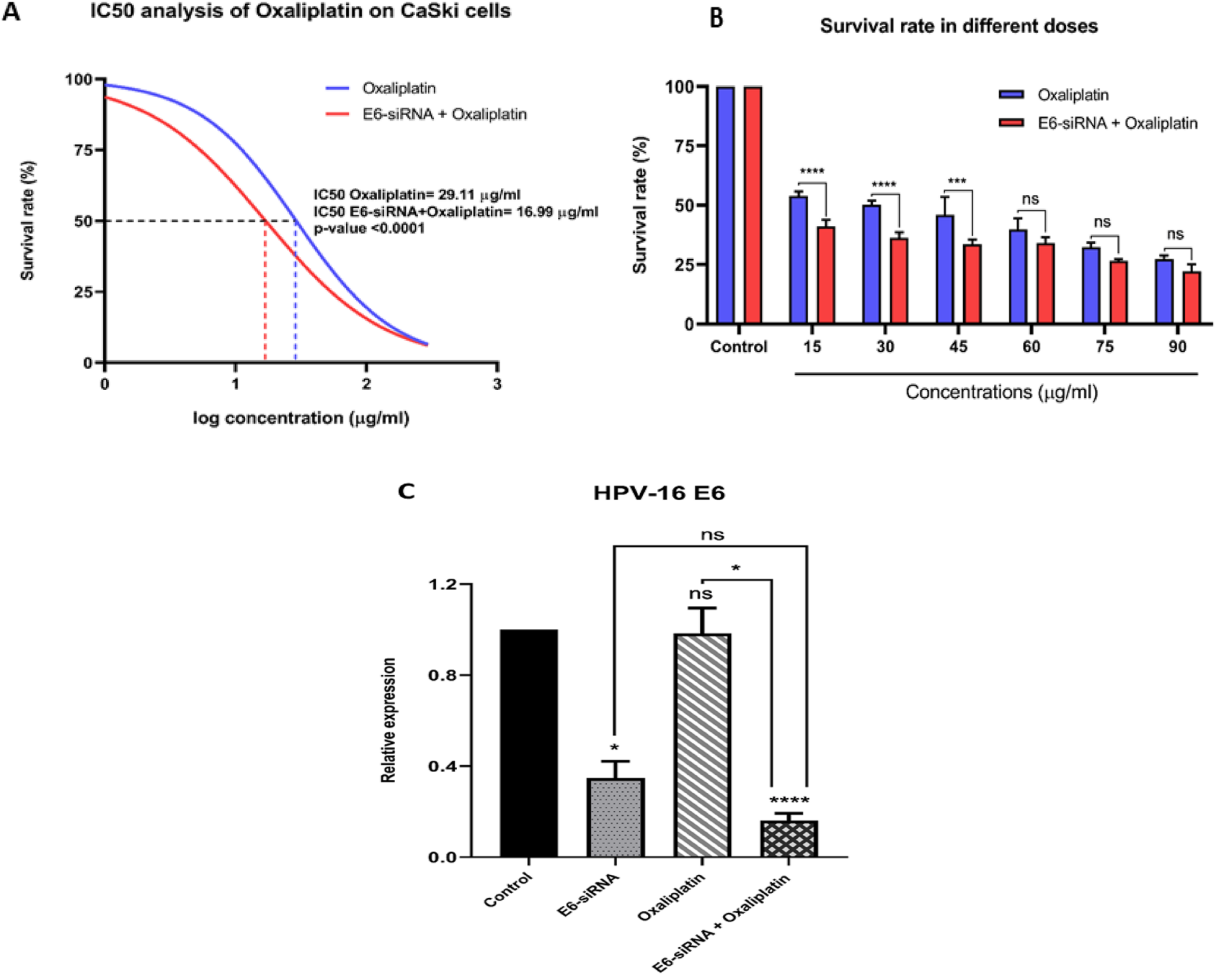
The effect of combining E6-siRNA and Oxaliplatin on the viability of CaSki cancerous cells and E6 expression. **A**, **B**. In the Oxaliplatin and siRNA combination, the drug’s IC_50_ was reduced compared to only chemotherapy with Oxaliplatin. **C**. The expression rate of E6 oncogene in combination therapy decreased significantly compared to other groups (*p < 0.05, **p < 0.01, ***p < 0.001, ****p < 0.0001)

MTT assay was used to evaluate the viability and proliferation of CaSki cells and determine the IC_50_ value of Oxaliplatin after treatment with Oxaliplatin alone or in combination with E6-siRNA at different doses. It was observed that after 24 h of incubation, treatment with Oxaliplatin alone had just approximately 50% effect on cell survival or proliferation with 29.11 µg/ml concentration compared to the control group (Fig. 2A). Moreover, in qRT-PCR analysis, a single treatment with either Oxaliplatin or E6-siRNA inhibit gradually E6 oncogene expression compared with the control group (Fig. 2C). On the other hand, the results showed that E6-siRNA transfection reduces the IC_50_ value of Oxaliplatin (16.99 µg/ml). In other word, it was indicated the concentration of Oxaliplatin was reduced to half of the initiate amount; however, the survival rate was almost a mere under 50% (Fig. 2B). It can be concluded that E6-siRNA especially increases the sensitivity of cancerous cells to the Oxaliplatin chemotherapy drug and subsequently lead to drug effective dose concentration.

### Knockdown of E6 in combination with Oxaliplatin could sensitize the apoptosis of CaSki cells

**Fig. 3:**
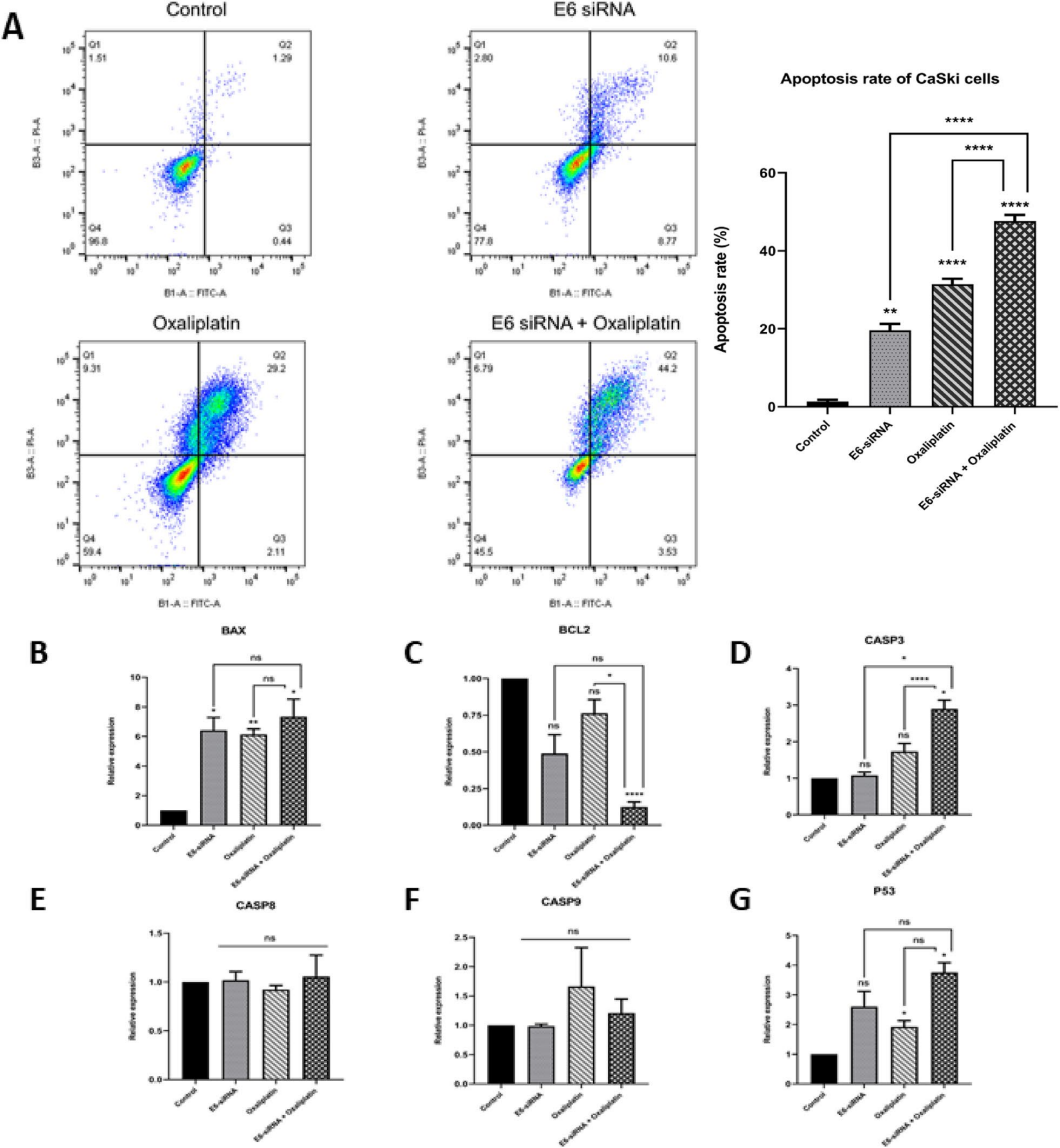
The effect of E6-siRNA and Oxaliplatin combination on the cell apoptosis. **A**. E6-siRNA transfection increases the sensitivity of CaSki cells to Oxaliplatin. **B**, **C**. *BAX* and *BCL2* expression levels increase and decrease due to combination therapy compared to control group, respectively. **D**. The *Casp-3* expression had remarkably increased following combination therapy compared to monotherapy with either Oxaliplatin or E6-siRNA. **E**, **F**. The *Casp-8* and *Casp-9 *expression resulting from combination therapy did not change significantly compared to other groups. **G**. The expression level of the *p53* gene as a result of combination therapy showed a dramatic change compared to other genes involved in apoptosis and other groups (*p < 0.05, **p < 0.01, ***p < 0.001, ****p < 0.0001)

The results of Annexin V/PI staining showed that the combination of Oxaliplatin and E6-siRNA, compared with the use of each alone, has a significant effect on inducing apoptosis and apoptotic genes. In other words, following the lonely use of either Oxaliplatin or E6-siRNA, the induction rate of apoptosis was 29.2% and 10.6%, respectively. However, the induction rate of apoptosis following Oxaliplatin and E6-siRNA combination therapy was 44.2% (Fig. 3A). Also, the investigation of apoptotic genes expression using qRT-PCR assay showed that the *p53* expression in combination therapy, compared to their sole use, has a significant promotion (Fig. 3G). On the other hand, caspase genes, such as *Casp-8* and *Casp-9,* did not show significant changes in combination therapy compared to the other two groups (Oxaliplatin or E6-siRNA monotherapy); however, the *Casp-3* had acceptable alters compared to other caspases and groups (Fig. 3D–F). Besides, apoptosis-associated genes, such as *BAX* and *BCL2*, have increased and decreased, respectively (Fig. 3B, C).

### Combination of E6 siRNA/Oxaliplatin arrested CaSki cells in the sub-G1 stage of cell cycle

**Fig. 4:**
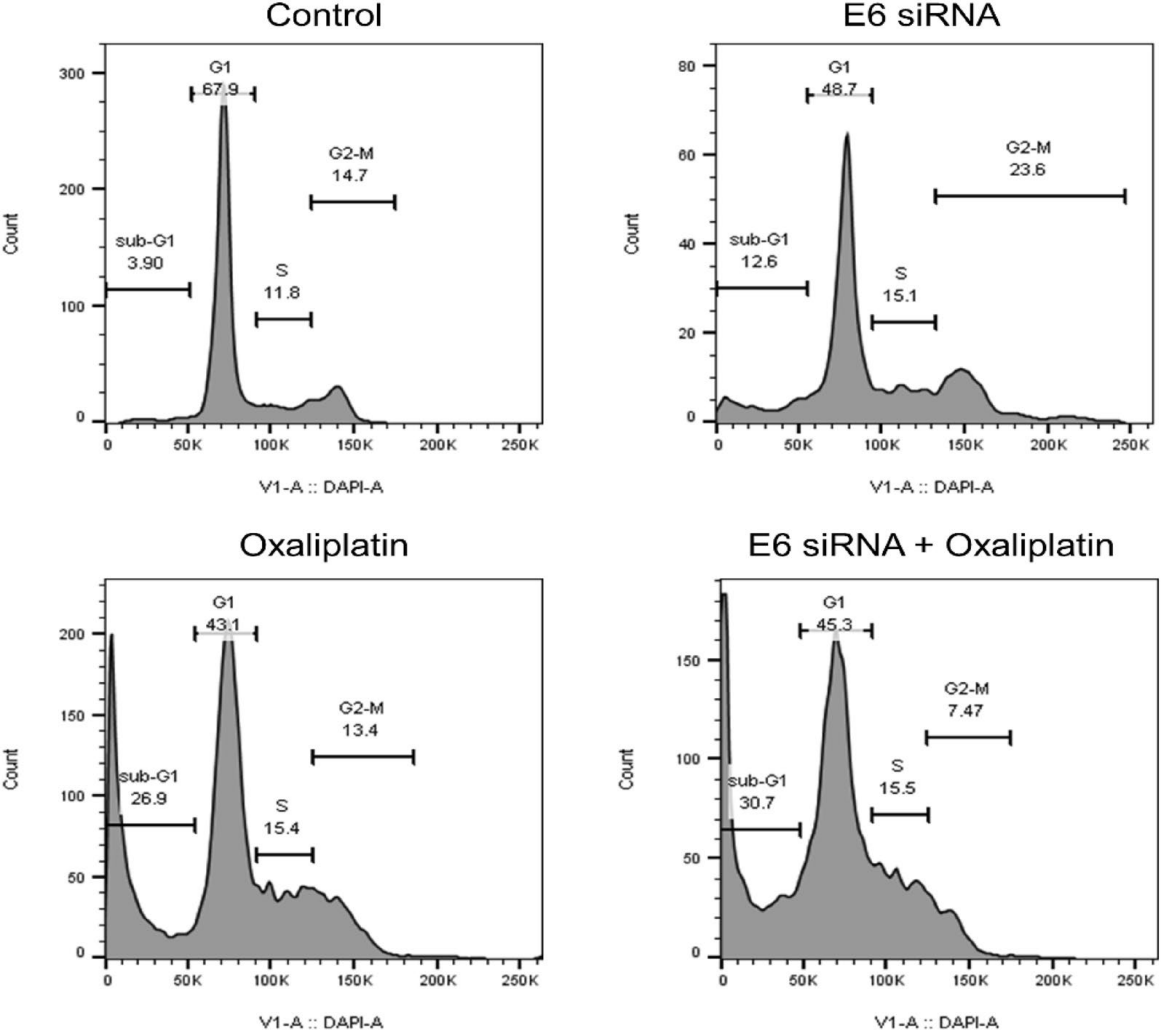
The influence of E6-siRNA combination with Oxaliplatin on the cell cycle. As a result of the siRNA with Oxaliplatin combination therapy and the knockdown of the E6 gene, the cell cycle is arrested in sub-G1 stage

To further evaluate the combination of Oxaliplatin and E6-siRNA effects on cell proliferation, we examined cell cycle distribution after siRNA transfection and Oxaliplatin treatment. Flow cytometry data have indicated that the cell population in the sub-G1 stage, following the use of Oxaliplatin and E6-siRNA alone, increased from 3.9 to 26.9% and 12.6%, respectively (Fig. 4). However, cell populations increased from 3.9 to 30.7% during the combination of E6-siRNA and Oxaliplatin. This study showed that Oxaliplatin and E6-siRNA combination therapy increases in the cell population in the sub-G1 stage and subsequently supports the arrest of the cell cycle in the sub-G1 stage.

### E6-siRNA combination with Oxaliplatin decreased stemness features in CaSki cells

**Fig. 5:**
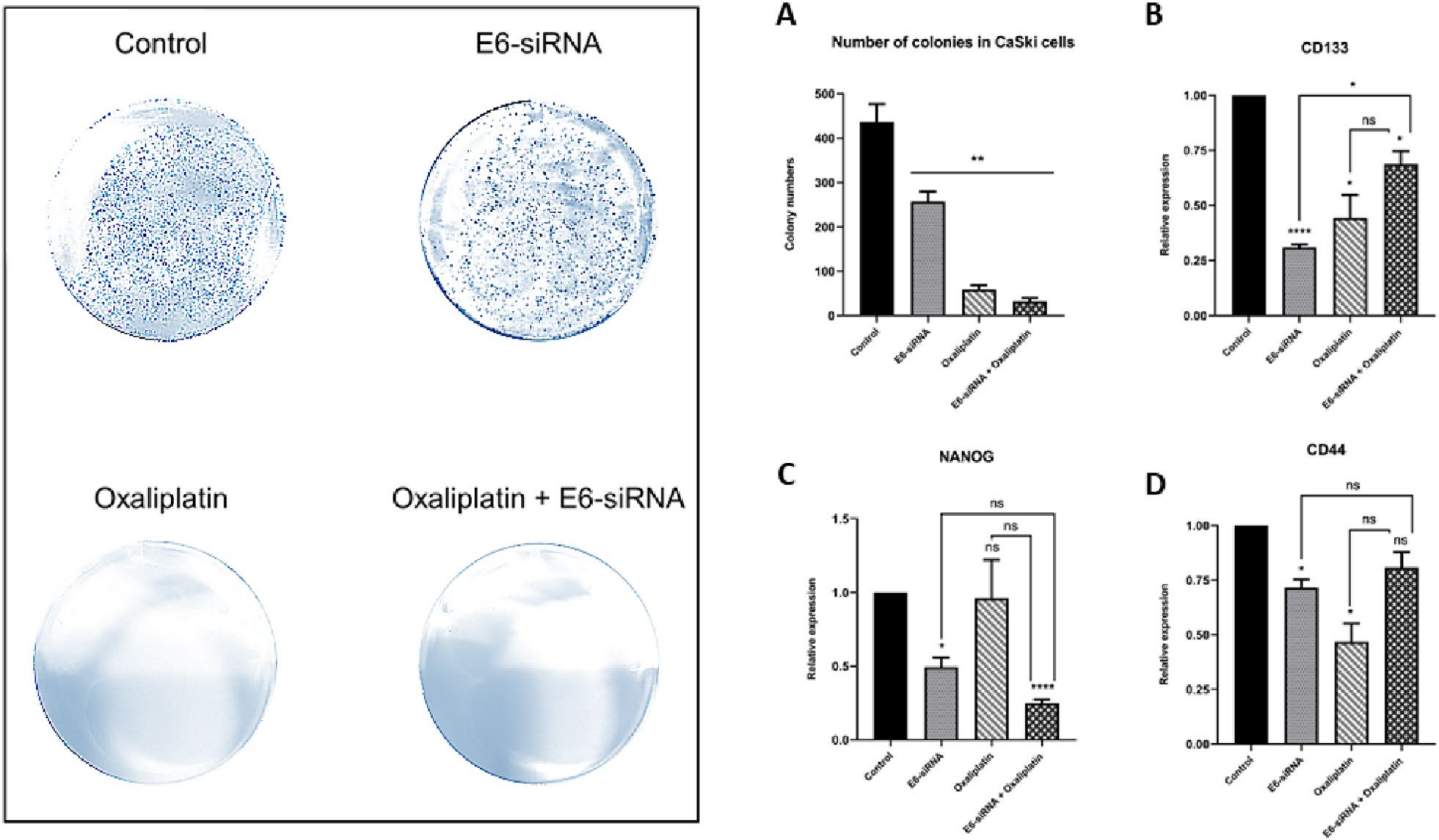
The effect of E6-siRNA and Oxaliplatin combination on stemness features. **A**. In the combination group, the number of colonies decreases significantly compared to control group. **B**, **D**. In the combination group, stem cell markers (*CD133* and  *CD44*) had shown slight changes compared to the other groups. **C**. In the combination group, the expression level of the *Nanog* stemness gene showed significant changes compared to the other groups, and the number of colonies altered depending on the Nanog pathway (*p < 0.05, **p < 0.01, ****p < 0.0001)

Colony formation assay was used to evaluate the effect of Oxaliplatin and siRNA combination on stemness properties. The results showed that, following Oxaliplatin and E6-siRNA combination, the number of colonies was significantly reduced compared to other groups (Fig. 5A). To support these results, qRT-PCR was used to evaluate the expression of stemness-associated genes. In this regard, it was shown that the expression of *CD44* and *CD133* genes in the E6-siRNA and Oxaliplatin combination group did not show significant changes compared to either the E6-siRNA or Oxaliplatin groups (Fig. 5B and D). In contrast, the *Nanog* expression in the E6-siRNA and Oxaliplatin combination group showed significantly alters compared to the control group (Fig. 5C).

### E6-siRNA combination with Oxaliplatin inhibits migration and related genes

**Fig. 6:**
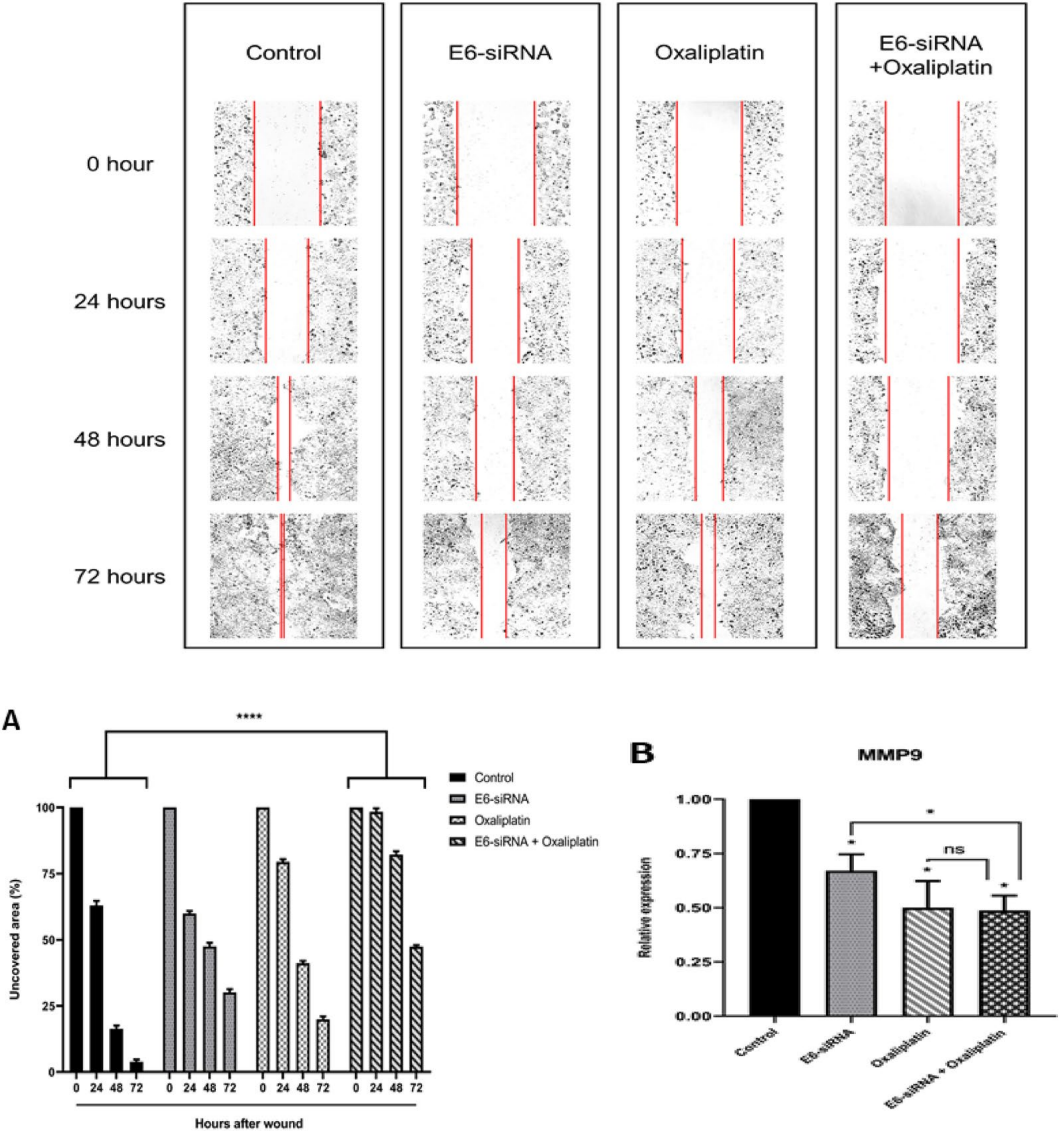
The effect of combining E6-siRNA with Oxaliplatin on CaSki cells migration and metastasis. **A**. Combination therapy with Oxaliplatin and E6-siRNA after 72 h leads to a significant reduction in the metastatic feature of CaSki cancerous cells compared to the control group. **B**. The expression of migration-related genes, such as *MMP9*, in the combination group showed a significant decrease compared to other groups (*p < 0.05, ****p < 0.0001)

According to the wound healing assay results, it was observed that the sole use of either E6-siRNA or Oxaliplatin could have a moderate reductional effect on cell migration. However, the Oxaliplatin and siRNA combination was associated with a significant reduction in CaSki cell migration compared to the control group after 72 h (Fig. 6A). In this regard, the effect of E6-siRNA and Oxaliplatin combination on the expression of migration-related genes, such as *MMP9*, was investigated by qRT-PCR (Fig. 6B). The results showed that the expression of *MMP9* decreased due to the use of E6-siRNA or Oxaliplatin alone. Still, following combination therapy, we significantly reduced the expression of *MMP9* with a less effective dose.

## Discussion

High-risk variants of HPV, such as HPV-16 and -18, can cause cancer and malignancy due to their oncoproteins’ affinity to substantial cellular tumor suppressor proteins, such as p53 and retinoblastoma protein [3]. Given the role of HPV in cervical cancer and other genital diseases, the development of appropriate methods for diagnosing, treating, and preventing this disease is needed. Surgery is recommended to treat cervical cancer at the early stages to remove cancerous tissue, sometimes accompanied by chemotherapy or radiation therapy [3]. However, surgery can sometimes be associated with infertility. On the other hand, radiation therapy is suitable for non-metastatic cancers, but in cases in whom the treatment is ineffective or the tumor is metastatic, the next step is chemotherapy [13]. Unfortunately, chemotherapy leads to a marked increase in drug resistance, poor pharmacokinetics of chemotherapeutic agents, and systemic toxicity due to a lack of specificity [14]. Also, tumor cells resistant to a chemotherapy drug are resistant to other chemotherapy drugs because they escape drug-induced apoptosis, activate DNA repair, and reduce drug uptake [15].

Since E6 oncogenes are expressed only in cervical lesions and are not found in normal tissue [16], targeting only E6 gene expression as a therapeutic target can be an appropriate strategy to prevent cervical cancer progression and normal tissue damage. Moreover, it has been reported that the p53 gene, the primary tumor suppressor gene, has been targeted in gene therapy, which can be combined with other methods, such as chemotherapy [17]. So, gene therapy with anti-cancer medications is an effective therapeutic method. In this study, we investigated the effect of the combination of E6-siRNA and Oxaliplatin on cervical cancer CaSki cells for the first time. The present study suggests that E6-siRNA inhibits the growth and proliferation of tumor cells in a dose- and time-dependent manner.

Moreover, according to the viability and cytotoxicity assay, it is speculated that E6-siRNA increases the susceptibility of malignant cells to Oxaliplatin and decreases the drug’s IC_50_ (29.11 µg/ml) to 16.99 µg/ml. Also, E6-siRNA can specifically promote the drug’s efficacy and stimulate cell death of the CaSki cancerous cell line through the p53-dependent apoptotic pathway. Evaluated E6 gene expression confirmed that the following monotherapy with either E6-siRNA or Oxaliplatin, the expression of the E6 gene decreases compared to the control group; however, the expression of this oncogene is significantly reduced during combination therapy. Besides, the expression of the p53 tumor suppressor gene and induction of apoptosis increased dramatically due to plummeted E6 oncogene expression. Several studies have been conducted to estimate the E6 oncogene knockdown efficiency in drug resistant. In 2011, a study conducted by Jung and Xin reported that the incidence of resistance to chemotherapy drugs following combination with siRNA was significantly reduced. Moreover, they showed that combination therapy was extremely effective than siRNA or drug monotherapy [18].

In 2018, H. Javadi et al. during a study on CaSki cancer cells showed that following combination therapy with E6-siRNA and cisplatin, the cells’ sensitivity to cisplatin and subsequent induction of apoptosis increases [19]. In the current study, the results of the MTT assay were also confirmed by flow cytometry assay with Annexin V/PI staining. It has been shown that monotherapy with either Oxaliplatin or E6-siRNA leads to lower induction of apoptosis than combination therapy. On the other hand, the combination of E6-siRNA with Oxaliplatin exponentially induces a p53-dependent apoptosis pathway, and the expression of *BCL2* and *BAX* genes plummets and elevates, respectively. In other words, transcriptional activation of the *BAX* gene is increased following the *p53* expression in tumor tissue and leads to the induction of apoptosis by chemotherapy in cervical cancer cells [20]. It can be concluded that, following combination therapy with Oxaliplatin and E6-siRNA, p53-dependent apoptosis and caspase-independent pathway induce. Moreover, the *BAX* gene expression is increased by *p53* and plays a vital role in p53-dependent apoptosis [21].

In the present study, cell cycle assay analysis demonstrated that the number of cells has increased in the sub-G1 stage after combination therapy with E6-siRNA and Oxaliplatin; subsequently, the cells accounted for in the G2-M phase were reduced by 50% compared to the control group. p21 expression has been reported to be directly related to an increase in p53. In this regard, p53-dependent expression of p21/WAF leads to cell cycle inhibition by cyclin protein-dependent kinases in the G1 phase and increases *BAX*. Increased *BAX* expression induces voltage-dependent anion channel activity, cytochrome c release, and cleavage of pro-caspases 3 and 9 [22]. On the other hand, as in our study, a decrease in the expression of anti-apoptotic factor *BCL2* indicates the induction of apoptosis by the p53-dependent internal mitochondrial pathway. According to the results, due to the increased sensitivity of cancer cells following E6-siRNA transfection, it is speculated that E6-siRNA reduces Oxaliplatin drug resistance and increases its effect on apoptosis, and cell cycle, and consequently reduces drug side effects.

One of the significant results in this investigation is a considerable reduction in the stemness characteristic and colony formation of CaSki cancerous cells following therapy with E6-siRNA and Oxaliplatin and consequently plummet in the expression of stemness genes, such as *CD133*, *CD44*, and *Nanog*. CD133 has been the first potential cancer stem cells (CSC) marker in human cervical cancer, and also it has been reported that the CD44 marker plays a critical role in enriching the cervical CSC population [23]. One of the essential factors for stem cells is Nanog, which is involved in angiogenesis, multidrug resistance, lymph node metastasis, immune resistance, malignant progression, and invasion [24]. In this regard, our study’s results indicate that the colony formation rate following combination therapy with E6-siRNA and Oxaliplatin was significantly reduced compared to the control group. Besides, the qRT-PCR assay showed that the expression of stemness-related genes decreased after combination therapy. So, *CD133* and *CD44* levels decreased compared to the control group; however, the expression of *Nanog* showed a significant decrease compared to other groups. As a result, it is hypothesized that the Nanog-dependent pathway induces colony formation reduction in combination therapy. Indeed, Nanog binds to the c-Jun promoter to increase c-Jun expression, which increases the expression of E6/E7 oncoproteins. In this regard, Díaz-Tejeda et al. in 2021 reported that Nanog regulates E6 and E7 expression by binding to activator protein-1 (AP-1) [24]. In other words, the expression and activity of *c-Jun* are directly related to Nanog. The c-Jun oncogene binds to DNA with high affinity as a heterodimer, causing the formation of AP-1 [25]. As a result, the activity of hr-HPV LCRs and the expression of E6 and E7 oncoproteins increased following the formation of AP-1 [26, 27]. Therefore, Díaz-Tejeda et al. showed that Nanog, as a transcription factor in stem cells, increased the activity of viral transcription regions and the expression level of E6/E7 oncogenes in cervical cancer cells [24]. On the other words, it has been reported that Nanog is involved in cervical cancer progression along with hr-HPV E6 and E7 oncoproteins as an essential factor for stem cells [24]. According to the results of our research, treatment with E6-siRNA and Oxaliplatin resulted in a significant reduction in Nanog expression consequently plummeting the ability of colony formation, metastasis, and eventually malignancy.

Metastasis and cervical cancer invasion are complex processes [2]. For example, HPV-16 E6 oncoprotein, following a decrease in Na + /H + exchanger regulatory factor 1 (NHERF1), increases ACTN4 actin cytoskeletal protein level and subsequently enhances actin polymerization, migration, and invasion of cervical cancer cells [28]. In this base, Zhang HR et al. in 2018 showed that the expression of *myosin 1b* and consequently *c-MYC* is increased by HPV16 E6/E7 oncoproteins and leads to the expansion of metalloproteinase matrix 1 and 9 (*MMP1* and *MMP9*) activity, destroy the extracellular matrix and ultimately improves the invasion of CaSki cancerous cells [28]. Furthermore, it has also been shown that proteolytic enzymes, such as MMP2 and MMP9, play an essential role in destroying the extracellular matrix during metastasis [29]. In the present study, the wound healing assay results indicate a significant reduction in cancer cell migration and subsequent lower decrease in wound size following siRNA and Oxaliplatin combination therapy. Although the scratching amount was acceptable in treatment with siRNA or Oxaliplatin alone, combination therapy showed a significant effect on cell migration after 72 h compared to the control group. Also, the results of our study and Asadzadeh et al. in 2021 showed that the amount of *MMP9* as a result of combination therapy has a significant reduction compared to both the control group and monotherapy in cancer cell migration [10]. As a result, it is speculated that by inhibiting the expression of E6 oncogene in combination therapy, the expression and activity of *MMP9* decreases and subsequently inhibits extracellular matrix degradation and CaSki cancerous cell metastasis.

## Conclusion

In summary, our study showed that specific inhibition of E6 oncogene expression by siRNA increased the susceptibility of cervical cancer cells to Oxaliplatin, thereby increasing the activity of apoptosis-related fractions and reducing cell proliferation and migration. It is also suggested that siRNAs can be used as a therapeutic strategy to increase the specificity and sensitivity of cancer cells to chemotherapy, influence the chemotherapy drugs, and subsequently reduce drug resistance in the treatment of cervical cancer. However, limitations, such as the siRNA transmission system and its half-life promotion in circulating, can be improved by identifying mechanisms.

## Data Availability

The data that support the findings of this study are available from the corresponding author upon reasonable request.
